# Collective quench dynamics of active photonic lattices in synthetic dimensions

**DOI:** 10.1038/s41567-025-02880-2

**Published:** 2025-05-01

**Authors:** Alexander Dikopoltsev, Ina Heckelmann, Mathieu Bertrand, Mattias Beck, Giacomo Scalari, Oded Zilberberg, Jérôme Faist

**Affiliations:** 1 Institute of Quantum Electronics, ETH Zürich, Zurich, Switzerland; 2 Quantum Center, ETH Zürich, Zurich, Switzerland; 3 Department of Physics, University of Konstanz, Konstanz, Germany

**Keywords:** Nonlinear optics, Ultrafast lasers

## Abstract

Photonic emulators have enabled the study of many solid-state and quantum optics phenomena, such as Anderson localization, topological insulators and non-Hermitian dynamics. Current photonic emulators are generally limited to bosonic behaviour with local interactions, but the use of synthetic dimensions offers a pathway to overcome this constraint. Here we investigate the flow of liquid light in modulated fast-gain ring lasers, and we establish a platform for emulating quench dynamics within a synthetic photonic lattice with equal densities across the reciprocal space. We apply an artificial electric field to the lattice and introduce a slow timescale to the flow, given by Bloch oscillations. Despite the dispersion and dissipation in our system, which desynchronize the Wannier–Stark ladder states, we were able to directly observe coherent oscillations facilitated by the fast gain. Additionally, we quenched a steady state of a coupled system onto an uncoupled one, which revealed coherent interactions between the decaying modes. These coherent dynamics resulted from the liquid state of light, which rapidly suppressed fluctuations at the shortest timescale of the system. This platform enriches our understanding of collective dynamics in the non-perturbative regime and improves our ability to control and generate coherent, multi-frequency sources.

## Abstract (web summary)

Synthetic dimensions allow photons and gauge fields to interact in photonic emulators. Now a study with fast-gain lasers shows that gain-driven coherence enables robust light flow in frequency space, establishing it as a viable platform for lattice emulation.

## Main

Analogue computing aims to replicate complex phenomena without any direct interaction with the original system. Fundamentally, a successful emulator requires tunable control operations as well as control over the initial state of the simulation. Photonic emulators have proven valuable for probing diverse solid-state phenomena to reveal intricate quantum and classical dynamics. These systems leverage the extended coherence time of light, which is enabled by typically weak interactions with the environment, to emulate phenomena such as Anderson localization^1^, topological insulators^2^, anyonic statistics^3^, fractional Bloch oscillations^4^, Thouless pumping^5–7^, moiré lattices^8^ and Landau levels^9^ in a controlled and adjustable experimental setting. These emulators not only broaden the intuition for solid-state dynamics but also push beyond known physical models by introducing non-Hermitian dynamics^10^ and enabling higher-dimensional studies^11^. They further offer the potential to engender new technologies and applications, for example, in quantum information processing^12^ or laser science^13–17^.

Most photonic emulators are realized through spatially varying structures in real space, which restricts the dimensionality, the available coupling schemes and the types of accessible nonlinearities^11,18,19^. Synthetic dimensions—constructed using properties like the time delay^18^ or the wavelength^11,19^—allow us to overcome these fundamental limitations^20^. Using artificial lattices, it is, for example, possible to increase the dimensionality of quantum systems^21^, such as photonic crystals^22^ and topological matter^11,23^, or even to control the non-Hermitian properties to demonstrate parity-time-symmetric ballistic to diffusive transitions^24^ or the funnelling of light^25^.

The notable coherence of light, which stems from its bosonic nature and its limited inherent interaction with external fields, complicates the study of particle interactions in photonic emulators. Nevertheless, interactions with gauge fields can be artificially created by designing a synthetic space^20^. For charge-like behaviour, it is possible to introduce artificial magnetic and electric fields in the synthetic space for photons^19,26^. For example, coupling multimode resonators under collective resonant modulation led to two-dimensional synthetic lattices in which the interleaving of the modulation phase induced time-reversal symmetry breaking and the appearance of topological edge states^27^. Similar techniques have been applied to cold atoms in synthetic lattices of neutral particles^11,28,29^. Other researchers have generated linear electrical gauge fields that induced Bloch oscillations^19,30,31^ through potential gradients within the lattice^32–34^.

Despite extensive research into light-coupling and gauge fields, collective lattice dynamics in synthetic dimensions driven by nonlinear interactions remain underexplored. Recent work studying solitons has demonstrated that exposure to a linear gauge field in synthetic space preserves oscillatory motion due to long-range interactions caused by a local nonlinearity in real space^35^. This behaviour differs from that of short-range nonlinearities, which are typically caused by the Kerr effect in the same space of the wave dynamics, where Bloch oscillations experience self-focusing or self-defocusing^34^, thus distorting the oscillation process. In another and recent example, a fast-gain laser led to the preservation of quantum-walk dynamics in synthetic space, leading to a ballistic expansion and stabilization of the laser spectrum facilitated by the long-range nonlinearities of these types of devices^36^. In these key examples, the comprehension and use of nonlinear phenomena in photonic lattices was critical, not only for advancing our understanding of the naturally interacting particles in lattices^37,38^ but also for optical technologies^16,17^. Interestingly, owing to the nonlinearities and the dispersed nature of photons in materials, these works illustrate the flow dynamics of light^39^ in frequency lattices^40–42^.

Here, we investigate the flow of a liquid phase of light in modulated fast-gain lasers and demonstrate that the associated synthetic frequency space acts as a photonic lattice emulator with a coherent flow. The liquid property of the light, enabled by fast-gain saturation, equalizes the population of the reciprocal space of the lattice and suppresses fluctuations, allowing us to study the quench dynamics of the flow at several timescales. For this purpose, we applied a detuned modulation that induced lattice dynamics with an artificial electrical field in the synthetic space, which defined the period of a Bloch oscillation. The varying on-site potential, set by dispersion and gain curvature, acted faster than this period and corrupted the expected oscillations. Crucially, however, we showed that the periodic dynamics persisted due to an even faster mechanism, namely, gain saturation. In addition, we quenched a broad state onto an uncoupled lattice with losses to reveal sustained coherence as the fast gain counteracted the dissipation. We attributed both the coherent flow of the oscillations and the coherent decay to the liquid phase of light in fast-gain lasers. This new active photonic emulation method broadens our understanding of collective dynamics in crystals and paves the way to optical devices with dynamical outputs at different timescales.

**Fig. 1:**
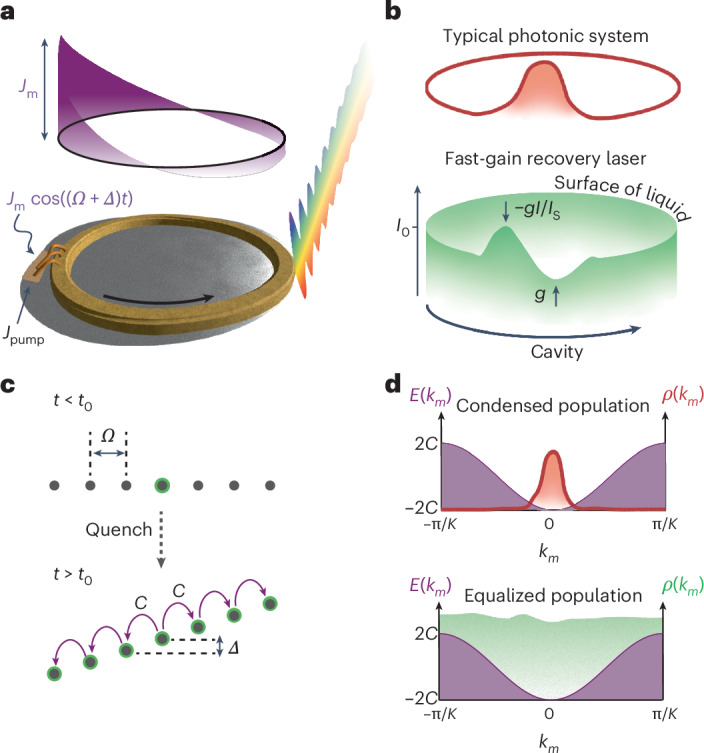
The fast-gain laser as a platform for realizing an active lattice in a synthetic dimension. **a**, Fast-gain ring laser. It is electrically pumped and has a section that is electrically modulated near the resonance frequency of the cavity *Ω*, with detuning *Δ* and depth *J*_m_, which emits a multimode spectrum (multicolour beam, cf. equation (1)). The modulated section drives a standing electromagnetic wave (purple), which translates through the gain to resonant phase modulation. **b**, Typical photonic state (red) after condensation to the bottom of the energy band, for example a pulse, and the steady state of a fast-gain modulated laser (green). The intensity in a fast-gain laser is clamped to a non-zero constant value, as every fluctuation in the intensity is suppressed by either gain *g* or gain saturation −*gI*/*I*_s_. This generates an artificial surface for the light, which is characteristic of liquids. **c**, Synthetic-dimension lattice composed of modes with free spectral range *Ω*. At time *t* = *t*_0_, the modulation is turned on, and the initial single-mode state (top, green) is quenched to modes that are parametrically coupled with effective hopping *C*. Detuned modulation leads to an on-site energy tilting (electric field) *Δ* (cf. equation (2)). **d**, The resulting band structure inherits its shape from the modulation. In the same space, the two photonic states from **b** are expected to produce different dynamics in their photonic lattices due to the nature of their stabilization mechanism. Typical non-Hermitian or nonlinear emulators will be local in momentum (top). The fast-gain platform has an equalized population in the reciprocal space (bottom).

The experimental platform of the emulator is a modulated ring-cavity quantum cascade laser (QCL) operating at a wavelength of 8 μm (Fig. 1a)^36^. The laser was processed using an inverted, buried InAlAs/InGaAs heterostructure fabricated by InP regrowth through metal–organic chemical vapour deposition, which ensured sufficient thermal and optical properties. d.c. electrical pumping was provided by wire bonds across the whole length of the device, which properly distributed the current. The a.c. signal was injected through a localized contact to ensure efficient a.c. modulation. The gain medium of the QCL, based on quantum-confined cascaded transitions, exhibited fast-gain saturation^43,44^, which suppresses intensity fluctuations around the steady-state intensity on subpicosecond timescales. In other words, the fast gain is a non-Hermitian and nonlinear interaction term that disfavours pulse formation, leading to a quasi-constant intensity ~*I*_0_ (Fig. 1b), such that phase rather than intensity variations govern the dynamics. Notably, the gain saturation was faster than any other contribution to the dynamics, creating a well-defined surface at *I*(*z*,*t*) ≈ *I*_0_ for the fluid phase, which gave liquid collective properties to the light in our emulator^39^. In the following, we present a full description of the underlying system that is subject to the fast gain.

We initialized the emulator to single-mode lasing using processing techniques that avoid backscattering and the associated nonlinear proliferation of modes^43^. At time *t*_0_, we turned the phase modulation on through radio-frequency (RF) injection with amplitude *J*_m_, which introduced dynamics into the system. When the modulation frequency matched the free spectral range of the cavity *Ω*/2π = 15.77 GHz, it induced coupling in a synthetic space spanned by the longitudinal modes of the ring (Fig. 1c)^11,26,45,46^. The resulting dynamics in the copropagating frame *z* relative to the initial lasing mode are given by the following modified complex Ginzburg–Landau equation^42^:

1 $$\begin{array}{l}\mathrm{i}\dot{E}=\mathrm{i}g\left(1-\frac{I}{{I}_\mathrm{s}}\right)E-\mathrm{i}\alpha E+\mathrm{i}\frac{1}{2}{g}_\mathrm{c}{\nabla}^{2}E-\frac{1}{2}\beta {\nabla}^{2}\\\qquad{E}+\varTheta \left(t-{t}_{0}\right)2C \cos \left({Kz}-\varDelta t\right)E,\end{array}$$

where *E* is the cavity field, *g* is the gain, *I* and *I*_s_ are the field and saturation intensities, *α* denotes the combined medium and waveguide losses, *g*_c_ and *β* are the quadratically approximated gain curvature and dispersion, respectively, and *K* = 2π/*L*, where *L* = 5.76 mm is the length of the cavity. The RF gain modulation translates to phase modulation through the linewidth enhancement factor^47,48^. Sufficiently, our model keeps only the phase modulation with depth 2*C* and resonant spatial frequency *K.* Also, *Δ* is the detuning relative to the free spectral range of the cavity and *Θ*(*t*) is the switch-on step function.

Although the complex Ginzburg–Landau equation provides a real-space description that is suited well for modelling the behaviour of the laser, intuitive insights into lattice dynamics in synthetic space can be achieved through a description in reciprocal space. Therefore, we use the well-defined spatial modes of the system with spacing *Ω* to write the cavity field in the modal basis *m* using *E* = ∑*A*_*m*_e^−i*mΔt* − i*mKz*^, where *A*_*m*_ are the mode amplitudes. By plugging this ansatz into the linear part of equation (1), so that we neglect the fast-gain saturation for now, we obtain the Hamiltonian for *t* > *t*_0_:

2 $$\begin{array}{l}{H}_\mathrm{lin}=\sum_{m}\left(V\left(m\right)-\mathrm{i}G{m}^{2}\right){a}_{m}^{\dagger}{a}_{m}\\\qquad+C\left({a}_{m-1}^{\dagger}{a}_{m}+{a}_{m+1}^{\dagger}{a}_{m}\right)+\mathrm{i}\left(g-\alpha \right){a}_{m}^{\dagger}{a}_{m},\end{array}$$

where the dispersion acts as an on-site potential energy *V*(*m*) = *Dm*^2^ + *Δm* and the gain curvature as on-site losses −*Gm*^2^, where *D* = *βK*^2^/2 and *G* = *g*_c_*K*^2^/2, the operators ${a}_{m}^{\dagger }$ and *a*_*m*_ denote the creation and annihilation of a photon in mode *m*, and *g* and *α* are the constant on-site gain and loss. We define the *m* = 0 mode as the initial lasing mode. At *t*_0_, we turned the modulation on and effectively induced nearest-neighbour coupling with amplitude *C* between the modes (Fig. 1c), which produced dynamics with a cosine band structure^19,30,48^. The role of the missing nonlinear part, the fast gain that leads to a quasi-constant intensity in the cavity space and the liquid properties, cannot be simply inserted into the Hamiltonian due to its nonlinear, dissipative nature. The fast gain can be understood as a mechanism that fills up the cavity space and, therefore, effectively equalizes the population of the reciprocal space of the synthetic lattice (Fig. 1d) where the band structure lies. This contrasts with existing photonic emulation systems, which would condense to the bottom of the energy band and produce dynamics governed by linear dissipation.

**Fig. 2:**
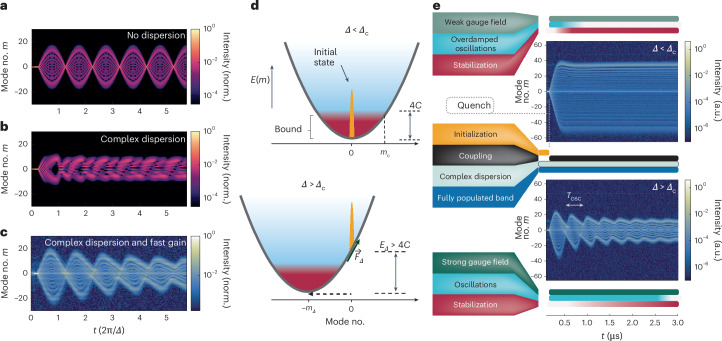
Enabling oscillations in complex dispersion through the fast gain, a tool for studying collective non-equilibrium dynamics. **a**, Simulation of the population in the synthetic space showing Bloch oscillations from a single-site excitation without complex dispersion (*D* = *G* = 0). **b**, Simulation with realistic complex dispersion (*D* = 0.0014*C* and *G* = 0.0077*C*). The oscillations are corrupted by the unequal energy spacing and selective dissipation. **c**, Experimental observation of collective quench dynamics in a biased active lattice with the same parameters as in **b** but with fast gain reviving the oscillations. **d**, At the moment of quenching, the initial state is at the central mode *m* = 0. When enabling resonant or near-resonant parametric coupling (*Δ* = 0 or *Δ* ≤ *Δ*_c_; top), we observed only bound spectral states due to the parabolic confining potential. For sufficiently large detuning (*Δ* > *Δ*_c_ and *E*_*Δ*_ > *E*_K_), the initial state excites modes mostly bound to the on-site energy, allowing the state to oscillate in the modal space. **e**, Measurement of dispersive Bloch oscillations and their stabilization. The left-hand column presents the functions and monitors of the emulator. On the right-hand side, we show the time-resolved dynamics in the active synthetic lattice for modulation detuning *Δ* below or above the critical value *Δ*_c_. In both cases, we initialized the system in a single synthetic lattice site. At time *t*_0_ = 150 ns, we quenched the state to a coupled and biased lattice with complex dispersion. Throughout the experiments, the fast gain nonlinearity forces the reciprocal space of the synthetic lattice to be full (quasi-constant intensity), leading to coherent dynamics. For modulated *Δf* = 1.22 MHz < *Δ*_c_ (top), the spectrum expanded ballistically, performed a damped oscillation and stabilized on a broad coherent spectrum. When *Δf* = 3.02 MHz > *Δ*_c_ (bottom), we observed oscillations with periodicity near *T*_osc_ = 2π/*Δ*, unlike the corrupted dynamics expected from systems with complex dispersion (cf. **b**). norm, normalized.

To study the flow of light in our system, we first manipulated the lattice dynamics by introducing off-resonant modulation, that is *Δ* ≠ 0, which tilted the on-site potential in the synthetic modal space, analogous to an electric field^19,31^ (Fig. 1b). In the absence of complex dispersion (*D* = *G* = 0), as the electric field increases, transport becomes less probable and the extended Bloch states morph into a Wannier–Stark ladder, where the electron trajectories become more localized^49,50^. In a semiclassical analogue, we can analyse the periodicity of the Bloch oscillations through *k*_*m*_, the reciprocal coordinate to the modal space *mK*, which experiences an effective linear force *F* such that $\dot{\left\langle {k}_{m}\right\rangle }=F=-\varDelta /K\to \left\langle {k}_{m}\right\rangle K={Kz}-\varDelta t$ (ref. ^51^). In the absence of dispersion, the band structure is *E*(*k*_*m*_) = 2*C* cos(*k*_*m*_*K*) (Fig. 1d), so that the motion in the frequency ladder would have an effective velocity $\dot{\left\langle {\rm{m}}\right\rangle }K=-2{CK}\sin \left(\left\langle {k}_{m}\right\rangle K\right)$, with a period of *T*_osc_ = 2π/*Δ* and oscillation amplitude 2*C*/*Δ*. Clearly, *k*_*m*_ is directly related to space in the corotating frame *z*, meaning that by choosing the RF modulation, we can directly dictate the shape of the band structure. Initialized in a single site, the reciprocal space would be fully populated and, therefore, the wavefunction would perform Bloch oscillations of the type presented in Fig. 2a. However, this coherence is disrupted when complex dispersion is introduced.

Dispersion introduces an approximately quadratic potential into the synthetic lattice, which impacts the coherence of the flow. This effect mirrors observations in doped semiconductors, where the Hartree potential arising from charges in the biased lattice breaks translational symmetry and, thus, requires a general eigenmode analysis^52^. The eigenstates of the linear Hermitian synthetic lattice (*G*, *g*, *α* → 0) reflect the competition between the parabolic confinement *Dm*^2^, and kinetic energy 4*C* (Supplementary Fig. 1). Note that the structure of the solutions remains qualitatively the same for any detuning *Δ*. Within the kinetic energy bandwidth, *E*_K_ = 4*C*, the excitations span the full extent of the parabolic confinement to form Hermite–Gauss type solutions. Above *E*_K_, the excitations are localized around the sites where the on-site energy overtakes the kinetic term. These states are like Wannier–Stark states, as they were approximately equidistant in energy and exhibit similar functional local forms shifted by the lattice site number. Initializing the system in a single frequency, overlapping with the Wannier–Stark-like ladder, we simulated these dynamics and found that without the fast gain, unequal energy spacings and mode lifetimes disrupted the ideal oscillatory dynamics (Fig. 2b).

Examining the same dispersed and non-Hermitian system in combination with the fast gain in our experimental platform yielded liquid dynamics that exhibited a fundamentally different result: the oscillations persisted despite the inherent dispersion (Fig. 2c). We studied the mode competition and stabilization using time-resolved spectral measurements (Methods), which captured the evolution of the liquid light in the active synthetic lattice. Thus, we revealed the unique impact of the fast-gain stabilization on the dynamics induced by the electrical field. For these measurements, we initialized the system in a single mode, and at time *t*_0_, applied modulation detuned from the RF resonance by *Δ*, effectively quenching the state. We first explore the moment of the quench. Recall that the initial state solely populated *m* = 0 with initial energy *E*(0) = 0 (Fig. 2d). The detuning *Δ* shifted the minimum of the potential by *m*_*Δ*_ = *Δ*/2*D* and lowered it by *E*_*Δ*_ = *Δ*^2^/4*D*, effectively providing the initial state at *m* = 0 with this relative energy *E*_*Δ*_. When this energy was smaller than the kinetic energy, the state fell into the Hermite–Gauss bound modes of the system (red region in Fig. 2d). The minimal detuning required to exceed this limit of *E*_K_ defines the critical detuning ${\varDelta }_\mathrm{c}=4\sqrt{{CD}}$. For small detunings, *Δ* < *Δ*_c_, the state rapidly expanded, which was followed by a damped oscillation and subsequent stabilization on a broad steady state (Fig. 2e, top). For sufficiently high detuning, *Δ* > *Δ*_c_, the state underwent periodic oscillations that stabilized coherently (Fig. 2e, bottom), unlike the expected decoherence in potentials with complex dispersion (Fig. 2b). Our simulations closely match the measurements (Supplementary Section 7), enabling us to extract the dispersion ~392 fs^2^ mm^−1^ (*D*_exp_ = 1.75 × 10^5^ rad s^−1^), phase modulation depth per unit time *M*_exp_ ≈ 2.47 × 10^8^ rad s^−1^ and the critical frequency detuning *Δf*_c_ = *Δ*_c_/2π ≈ 2.96 MHz. We ascribe the persistence of oscillations (Fig. 2c,e) to the fast-gain dissipative four-wave mixing nonlinearity (the term *g*(1 − *I*(*t*,*z*)/*I*_s_)*E* in equation (1)), which introduced long-range coupling in the frequency lattice. The well-defined surface generated at *I*_0_ indicates the incompressibility of the light in fast-gain lasers, in contrast with a compressible slow-gain system (Supplementary Fig. 6). This nonlinearity stabilized the system faster than the other timescale in the system^53,54^, that is oscillations or dispersion (Supplementary Sections 2 and 8), and gave the light the properties of a liquid flowing coherently in one dimension, which was notably visible in the presence of noise (Supplementary Fig. 7).

**Fig. 3:**
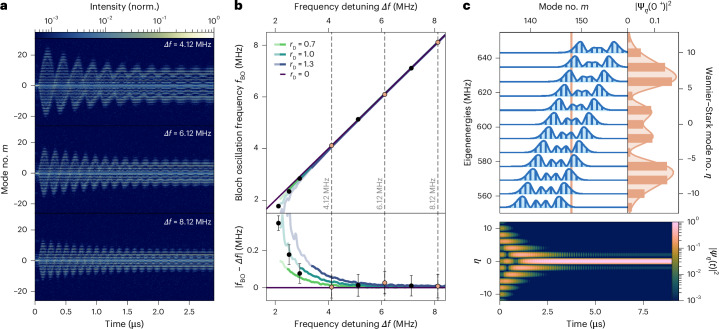
Observation of Bloch oscillations in the synthetic space emulator and their decay to a Wannier–Stark state. **a**, Time-resolved spectral measurement at three different detuning values, *Δf* = 4.12, 6.12 and 8.12 MHz. **b**, Oscillation frequency *f*_BO_ as a function of the detuning *Δf* (dots, top) and the oscillation frequency difference |*f*_BO_ − *Δf*| (dots, bottom) for values extracted from experiments (circles) with simulated results for various levels of dispersion (lines). The oscillation frequencies were extracted as the peaks of the Fourier transform of the evolution of the participation ratio over time. The error bars denote the width of a peak at 1% height reduction. The oscillation frequency deviates from the modulation frequency for small detuning values due to increasing dispersion in the Wannier–Stark ladder that stems from the quadratic potential. Here *r*_D_ denotes the ratio between the dispersion value we used in each case and the experimentally retrieved value. Bright colours indicate *Δ* < *Δ*_c_, and dark colours *Δ* > *Δ*_c_. **c**, The initial state (orange line), projected onto the Wannier–Stark supermodes (blue, left), decays over time to a single Wannier–Stark state (bottom). norm., normalized.

We analysed the oscillations and their stabilization rate after quenching to the dispersed Wannier–Stark ladder, where the mode structure shifted by *m*_*Δ*_. We compare several cases of spectral evolution with modulation detunings of *Δ*/2π = *Δf* = 4.12, 6.12 or 8.12 MHz. As the modulation frequency was increased, the period and amplitude decreased as expected (Fig. 3a). The oscillation period deviated from the ideal Bloch period *T*_osc_, primarily due to the quadratic dispersion (Fig. 3b). We found that relatively far from resonance, the oscillation frequency *f*_BO_ followed the detuning *Δf*. However, as the detuning approached the critical value *Δf*_c_, *f*_BO_ deviated from *Δf*, indicating the influence of the complex dispersion and nonlinear fast gain. The decay rates, extracted from the contraction of the broadest spectra in each Bloch oscillation (Supplementary Fig. 4), reveal that smaller detuning also resulted in a slower decay, whereas higher detuning led to a constant decay time (~1 μs) across all dispersion values. This means that although the gain was fast, the interplay between the gain and the dispersion was relatively slow, operating on a long (microsecond) timescale. The stabilization process can be attributed to the interplay between the gain curvature and the fast-gain saturation, which suppressed any intensity fluctuations in both space and time to favour a single, spatially extended, Wannier–Stark state (Supplementary Section 7). The stabilization dynamics in Fig. 3c shows the initial mode overlap of *ψ*(0^+^) for *Δf* = 8.12 MHz as well as the evolution of the projection of state *ψ*(*t*) onto the system modes and its decay to one Wannier–Stark-like state. During the decay process, the modes showed an interference pattern, indicating that there was a coherent interplay throughout. Thus, we have demonstrated that quenching our synthetic lattice to a Wannier–Stark ladder under a detuning-induced force led to coherent Bloch oscillations, even in the presence of complex dispersion. Enabled by the fast gain, these oscillations illustrate the resilience of the system to dissipation and dispersion, with their period scaling directly with the applied force, as expected for Bloch oscillations.

**Fig. 4:**
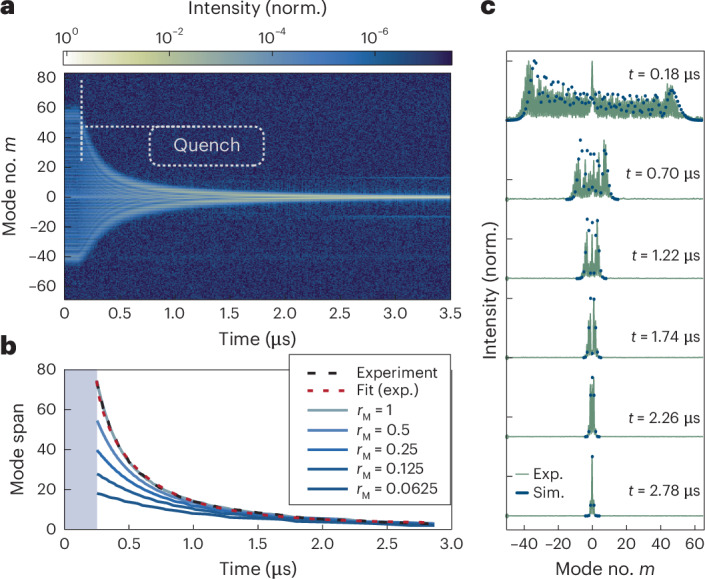
Decay of a broad spectrum when coupling was suddenly stopped. **a**, We initialized the lasing system in a state with broad occupation in the synthetic lattice and quenched it at *t* = 0.15 μs onto the uncoupled system. The initially broad lasing spectrum, without a broadening mechanism such as phase modulation, decayed into a single-mode state chosen by the gain curvature. **b**, Calculated bandwidth versus time for the measurement (with a fit) and simulations of the decay rates for different relative values of the modulation *r*_M_ = *M*/*M*_exp_ before the quench. **c**, Bessel-like spectra throughout the decay, in the experiment (green line) and simulation (blue dots) using equation (1). The fast gain governed the dynamics by keeping coherence between the modes until full decay, thus preserving a Bessel-like state. Exp., experiment; Sim., simulation.

We next examined the dynamics when coupling was removed, which revealed other coherence properties unique to the liquid phase in our emulator. We performed another time-resolved measurement to investigate the quench dynamics when the linear coupling was turned off and when the remaining interactions were solely nonlinear. We initialized the emulator in a state of very broad occupation of the synthetic lattice through resonant modulation, and then at time *t* = 0.15 μs, we rapidly turned off the modulation (Supplementary Section 6), effectively quenching the state to the uncoupled lattice (Fig. 4a). Without parametric nearest-neighbour coupling, the fast-gain nonlinearity was not sufficient to sustain this broad state and it decayed to a spectrally narrow state *δ*(*m*) (Fig. 4a). The decay times of the bandwidth were in the range 0.33–0.79 μs (Fig. 4b), signifying the long timescale in our system related to the interplay between complex dispersion and the fast-gain nonlinear interactions. Although the linear coupling between the modes was absent, the nonlinear long-range interaction induced by the fast-gain maintained coherent interference between modes to produce Bessel-shaped dynamics (Fig. 4c). This measurement demonstrates the unique role of the fast gain: by suppressing fluctuations, it maintains coherence between the modes throughout the whole evolution, even after substantial submicrosecond quenches, reinforcing the liquid properties of light in our emulator^57^.

In this work, we developed a synthetic-dimension platform using fast-gain saturation in real space to produce a liquid phase of light with an equalized population across the reciprocal space of the frequency lattice. This platform enables the study of collective quench dynamics in active lattices. By applying an artificial electric field, we demonstrated that robust coherent oscillations persist despite the competition with complex dispersion. Because of the liquid phase of the light, which stems from the fast gain, the oscillations are seen as the periodic population of sites at a period close to the Bloch frequency. We found that, unlike a linear and translation-invariant system, the oscillation frequency deviates from the Bloch frequency as the detuning is reduced. Over time, the oscillations decay on microsecond timescales to a single Wannier–Stark mode^50^. As a further application of the platform, we performed a quench from a coupled system to an uncoupled ‘flat band’ configuration, observing once more that the coherent flow overtakes dissipation despite the complex on-site potential. Based on these observations, we identified three distinct timescales in our system: (1) the longest is related to the Bloch oscillations, (2) the intermediate one to dispersion and linear dissipation corrupting the oscillations and (3) the shortest to fast-gain recovery, which enforces the liquid phase for the light and overcomes the deviations due to dispersion and dissipation.

By quenching the system, we were able explore the hydrodynamics of a one-dimensional, out-of-equilibrium, weakly interacting bosonic synthetic lattice, which can be compared to cold atomic experiments^55^. The coherent transformation during quenching relates this process to a liquid phase of light, such that small fluctuations decay faster than any other process. Intriguingly, the tendency of fast-gain systems to emit with a quasi-constant intensity mirrors the influence of the Pauli exclusion principle on a fermion population in a lattice, which inhibits condensation and the generation of optical pulses. Just as the fermionic nature of electrons compels them to populate the entire Brillouin zone, the fast gain discourages condensation of the light intensity and causes the state to span evenly over the whole intracavity period^56^. Thus, fast-gain lasers could be a highly adaptable platform for solid-state emulations of collective phenomena, particularly when interactions lead to equalization of the density in the reciprocal space. We posit that our platform may enhance our understanding of collective non-equilibrium dynamics within crystals and also inspire new multi-frequency photonic devices.

## Methods

To perform time-resolved measurements of the photonic state in the synthetic space, we relied on boxcar sampling of the output signal of a Fourier transform infrared (FTIR) spectrometer (Vertex 80, Bruker). At time *t*_0_, we turned on the RF electrical power injection (SMF 100A, Rohde & Schwarz combined with an RF amplifier) into the laser by triggering an RF switch (RFSPSTA0218G, RF-Lambda) using the UHF Zürich Instruments boxcar. This trigger also served as a time reference for the delay of the boxcar window. The boxcar labels sampled data as a function of the delay of the integration window. A time step of 5–10 ns was chosen between each integrating window. To limit the acquisition time but still provide the system with enough time for meaningful evolution, the trigger was set to repeat only every 20 μs. The scanning velocity of the FTIR spectrometer was set to 20 kHz in accordance with Nyquist criteria to avoid any aliasing effects. At the output of the FTIR spectrometer, we used a 20-MHz 3-dB-bandwidth photovoltaic mercury–cadmium–telluride detector (Kolmar Technologies). The temporal evolution was then reconstructed by binning the acquired samples from the FTIR spectrometer to different boxcar delays and FTIR spectrometer stage delays, and then Fourier transformed only with respect to the stage delay to form time-dependent spectra.

## Online content

Any methods, additional references, Nature Portfolio reporting summaries, source data, extended data, supplementary information, acknowledgements, peer review information; details of author contributions and competing interests; and statements of data and code availability are available at 10.1038/s41567-025-02880-2.

## Supplementary information

Supplementary Information Mathematical derivations, numerical study and Supplementary Figs. 1–9.

## Data Availability

The data that support the findings of this article are available in the ETH Research Collection at 10.3929/ethz-b-000682790.

## Code availability

Codes are available in the ETH Research Collection at 10.3929/ethz-b-000682790.
