# Supplementary material for: Collective quench dynamics of active photonic lattices in synthetic dimensions
Source: Nat Phys. 2025 May 1;21(7):1134–40. doi: 10.1038/s41567-025-02880-2 (PMC12263436; doi:10.1038/s41567-025-02880-2)
Supplement: Supplementary file 1 — Mathematical derivations, numerical study and Supplementary Figs. 1–9. [file 41567_2025_2880_MOESM1_ESM.pdf]

nature physics

Supplementary information

https://doi.org/10.1038/s41567-025-02880-2

Collective quench dynamics of active photonic lattices in synthetic dimensions

In the format provided by the authors and unedited

Table of Contents

1. Derivation of the coupled mode equations under detuned modulation 3

2. Eigenmode map and equal filling of the reciprocal space 4

3. Energy considerations in the synthetic space under detuned modulation 5

4. Analytical solution of steady states 6

5. Coherence of the final states 8

6. Experimental setup 9

7. Interplay between complex dispersion, artificial electric field and fast gain 10

8. Perturbation analysis 13

9. Dispersion of perturbations 15

References 16

1. Derivation of the coupled mode equations under detuned modulation

In this section we derive the linear equation of motion, in the absence of fast-gain. The linear and energy conserving part of the EM field equations in the rotating frame of the central mode is given by

$$\dot{E} = i\frac{1}{2}\beta\nabla^2 E - iMcos(Kz - \Delta t)E.$$

Using the modal decomposition $E(t,z) = \sum A_n(t)e^{-in\mathrm{Kz}}$

$$\sum \dot{A_n}(t)e^{-in\mathrm{Kz}} = -i\frac{1}{2}\beta K^2 n^2 \sum A_n(t)e^{-in\mathrm{Kz}} - i\frac{M}{2}\left(e^{i(Kz-\Delta t)} + e^{-i(Kz-\Delta t)}\right)\sum A_n(t)e^{-in\mathrm{Kz}}$$

$$= -iDn^2 \sum A_n(t)e^{-in\mathrm{Kz}} - iC\sum\left(A_n(t)e^{-i\Delta t - i(n-1)\mathrm{Kz}} + A_n(t)e^{\Delta t - i(n+1)\mathrm{Kz}}\right)$$

, where we defined $D = \frac{1}{2}\beta K^2$ and $C = \frac{M}{2}$.

Next, we derive the equation for mode m by projecting on $e^{-im\mathrm{Kz}}$ and integrating over the extent of the cavity:

$$\begin{aligned}\int \sum \dot{A_n}(t)e^{-in\mathrm{Kz}}e^{im\mathrm{Kz}}dz \\ = -iDn^2 \int \sum A_n(t)e^{-in\mathrm{Kz}}e^{im\mathrm{Kz}}dz \\ - iC\int \sum\left(A_n(t)e^{-i\Delta t - i(n-1)\mathrm{Kz}}e^{im\mathrm{Kz}} + A_n(t)e^{i\Delta t - i(n+1)\mathrm{Kz}}e^{im\mathrm{Kz}}\right)dz\end{aligned}$$

$$\rightarrow \dot{A_m}(t) = -iDm^2 A_m(t) - iC\left(A_{m+1}(t)e^{-i\Delta t} + A_{m-1}(t)e^{i\Delta t}\right).$$

We can further develop these equations, and use the ansatz $B_m = A_m e^{-im\Delta t}$ and substitute to have

$$\left(\dot{B_m} + im\Delta B_m\right)e^{im\Delta t} = -iDm^2 B_m e^{im\Delta t} - iC\left(B_{m+1}e^{i(m+1)\Delta t}e^{-i\Delta t} + B_{m-1}e^{i(m-1)\Delta t}e^{i\Delta t}\right)$$

and finally get

$$i\dot{B_m} = (Dm^2 + \Delta\mathrm{m})B_m + C(B_{m+1} + B_{m-1}).$$

This equation has the form of mode hopping with the stationary potential equal to $V(m) = Dm^2 + \Delta\mathrm{m}$. This set of equations can be upgraded to a quantum Hamiltonian by introducing the operators $a_m^\dagger, a_m$ for the creation and annihilation of mode m.

2. Eigenmode map and equal filling of the reciprocal space

The coupled mode equations that we derived in the previous section constitute the supermodes of the system (Fig. 1D). Fig. S1A shows the same modes presented in Fig. 1D in the main text, but as they are represented in the reciprocal space, which is also the cavity space. The purple area indicates the phase modulation, and consequently, the band structure of the system in the absence of dispersion. The depth of the modulation dictates the bandwidth of the band structure. The modes of the dispersed system can be roughly divided into two types. The first are Hermite-Gauss like solution that are both bound in the frequency space and the cavity space. These do not support periodic dynamics over the Brillouin zone. Their energies reside below the kinetic energy limit of $4C$. The second type are mode that are bound to the dispersion energy but fluctuate around it proportionally to the kinetic energy. These modes are two-fold degenerate, allowing for the construction of propagating modes which support semiclassical traveling along the Brillouin zone associated to the Bloch-oscillations.

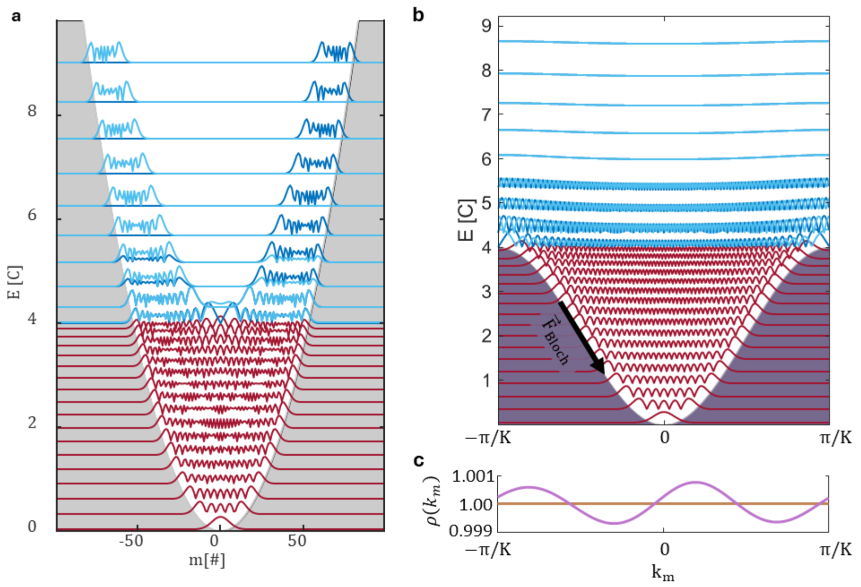

Figure S 1. The supermodes in the synthetic space, and the equal filling of the reciprocal space k_m. (a) The eigenmodes diagram in a quadratic potential induced by the dispersion, presented on a vertical energy scale; only every 4th mode is presented. Modes with $E > 4C$ become degenerate (marked with light and dark blue to differentiate the modes) but with opposite central momenta in the reciprocal space, taking the form of a Wannier-Stark ladder. (b) The same supermodes as in (a) represented in the reciprocal domain of the lattice, showing bound modes that correspond to the Hermite-Gauss like modes (red) and extended modes (blue) that support translation of the wavefunction over the reciprocal space, similar to the semiclassical description of the force in the Brillouin zone. Even when the system consists of dispersive modes and detuned modulation, the population of the reciprocal space stays equal. (d) The initial single mode state (gold) and the final (purple) densities of the light in the modulated fast-gain ring laser. The fast-gain is forcing equal population of the reciprocal space throughout the full dynamics of the system.

The interplay between these modes under quench dynamics is set by their dispersion, the gain curvature and the fast-gain. The later is responsible for stabilization of the intensity on a constant value around $I_0 = I_s\left(1 - \frac{\alpha}{g}\right)$, with the parameters taken from Eq. 1 in the main text. The fact that the full cavity is equally populated, along with the description of the system in the synthetic frequency lattice of the cavity, lead to the fast-gain being a mechanism that sets equal population of light in the reciprocal space of the synthetic lattice (Fig. S1B). This mechanism is responsible for all-time coherent quench dynamics.

3. Energy considerations in the synthetic space under detuned modulation

The potential we derived in the previous section, $V(m) = Dm^2 + \Delta m$, can be also written as

$$V(m) = \mathrm{D}\left[\left(m + \frac{\Delta}{2D}\right)^2 - \frac{\Delta^2}{4D^2}\right] = \mathrm{D}(m + m_0)^2 - E_\Delta$$

, which means that the $m = 0$ was shifted from the minimum of the potential by $m_0 = \frac{\Delta}{2D}$, and the minimum was reduced by $E_\Delta = \frac{\Delta^2}{4D}$, compared to $\Delta = 0$.

As we expect to observe Bloch oscillations only for unbound modes, we require the shift to be larger than the maximum kinetic energy of the bound states, meaning $E_k = 4C$. Therefore, the minimum force for Bloch oscillations is given by

$$\frac{\Delta^2}{4D} = E_\Delta > E_k = 4C \rightarrow |\Delta| > 4\sqrt{CD}$$

Moreover, in the absence of dispersion, we can find the exerted force in the synthetic space simply by

$$F = -\frac{dV}{d(mK)} = -2Dm/K - \Delta/\mathrm{K} \rightarrow \; F(D = 0) = -\Delta/\mathrm{K}.$$

Similar to $\dot{P} = F$, we consider this force as the change in k_m, which the reciprocal coordinate to the spatial frequency, that carries the units of space [1]. This leads to

$$\dot{k_m} = -\Delta/\mathrm{K} \rightarrow k_m K = Kz - \Delta t$$

, which exactly matches our starting point. With zero dispersion, the system is translationally symmetric in the frequency space. In that case, the energy of the bandstructure in reciprocal space is $E(k_m K) = 2C\cos(k_m K)$, and the motion in the synthetic lattice is given by

$$\langle \dot{m} \rangle K \propto \frac{\partial E}{\partial k_m} = 2CK\sin(\langle k_m \rangle K)$$

where the period is $T_{osc}|_{D=0} = \frac{2\pi}{\Delta}$. However, our dispersive system is not translationally symmetric in frequency space, and this period will change with increasing dispersion.

4. Analytical solution of steady states

We derive the steady state solution to the wave question with detuning $\Delta > \Delta_c$. We observe that these modes have almost constant intensity, non-zero phase modulation that follows the frequency of the modulation, and a small modulation of the intensity that follows twice the frequency of the modulation. We first derive the phase and then the correction for the amplitude.

a. Phase of steady states for $\Delta > \Delta_c$

The equation is

$$\dot{E} = \frac{1}{2}\left[g_0\left(1 - \frac{I}{Is}\right) - \alpha_w\right]\left(\frac{c}{n}\right)E + i\frac{1}{2}\beta\nabla^2 E + \frac{1}{2}g_c\nabla^2 E - iMcos(Kz - \Delta t)E$$

We assume FM laser state with $E = E_0 e^{i\phi x}$ ($x = Kz - \Delta t$), with quasi continuous amplitude. We get

$$-i\phi'\Delta E = \left[\frac{1}{2}\left[g\left(1 - \frac{|A|^2}{I_{sat}}\right) - \alpha\right] + \frac{i}{2}\beta(i\phi''K^2 - (\phi'K)^2) + \frac{1}{2}g_c(i\phi''K^2 - (\phi'K)^2) - iMcos(x)\right]E$$

The equation for the imaginary part is

$$-\phi'\Delta E = -\frac{1}{2}\beta(\phi'K)^2 E + \frac{1}{2}g_c\phi''K^2 E - Mcos(x)E$$

We assume that $\phi'\Delta \gg -\frac{1}{2}\left(\beta\phi'^2 + \frac{1}{2}g_c\phi''\right)K^2$ and get

$$\phi' = \frac{M}{\Delta}cos(x)$$

$$\phi = \phi_0 + \frac{M}{\Delta}sin(x)$$

Plugging this back to see if we indeed the approximation applies, we get

$$Mcos(x) \gg -\left(D\left(\frac{M}{\Delta}cos(x)\right)^2 + G\frac{M}{\Delta}sin(x)\right)$$

, where $G = \frac{1}{2}g_c K^2$. We find the requirement $\Delta \gg \sqrt{MD}$ which is fulfilled when $\Delta \gg \Delta_c$, and $1 \gg G/\Delta$ which is reasonable in our system because $\frac{G}{\Delta} < \frac{G}{\Delta_c} \sim 0.32$.

b. Amplitude of steady states

Assuming that the steady state carries the phase we derived in the previous section, we calculate the small change in amplitude that occurs in the intensity. We start again, but we assume that small changes in the amplitude are possible. We pre calculate the second derivative

$$\nabla^2 E = K^2(Ae^{i\phi})'' = K^2\left(A'^{e^{i\phi}} + iAe^{i\phi}\phi'\right)'$$
$$= K^2A''e^{i\phi} + i2K^2A'e^{i\phi}\phi' - K^2Ae^{i\phi}(\phi')^2 + iK^2Ae^{i\phi}\phi''$$

, and the full equation becomes

$$-i\phi'\Delta Ae^{i\phi} = \frac{1}{2}\left[g\left(1-\frac{|A|^2}{I_{sat}}\right)-\alpha\right]Ae^{i\phi} + \frac{1}{2}(i\beta+g_c)\left(K^2A''e^{i\phi}+i2K^2A'e^{i\phi}\phi'-K^2Ae^{i\phi}(\phi')^2+iK^2Ae^{i\phi}\phi''\right) - iMcos(x)Ae^{i\phi}$$

Comparing the imaginary and real parts we get the following:

1) The extended equation for the imaginary part is

$$-\phi'\Delta = \frac{1}{2}\left(\beta\frac{A(x)''}{A(x)}-\beta(\phi')^2+2g_c\frac{A'}{A}\phi'+g_c\phi''\right)K^2 - Mcos(x)$$

, where we already assumed that the change in the amplitude is very small compared to the amplitude itself.

2) The amplitude equation using the known phase is then given by

$$g\left(1-\frac{|A|^2}{I_{sat}}\right)-\alpha = \left(2\beta\frac{A'}{A}\phi'+g_c(\phi')^2+\beta\phi''-g_c\frac{A''}{A}\right)K^2$$

We assume the phase we found previously, and that $\frac{A''}{A},\frac{A'}{A} \ll 1$ to find

$$g\left(1-\frac{|A|^2}{I_{sat}}\right)-\alpha = 2\left(G\frac{M}{\Delta}\left(cos(x)\right)^2 - Dsin(x)\right)\frac{M}{\Delta}$$

We now define $f(x) = 2\left(G\frac{M}{\Delta}\cos^2(x) - Dsin(x)\right)$ and write

$$A = \sqrt{I_{sat}}\sqrt{1-\frac{\alpha}{g}}\sqrt{1+\frac{f(x)\frac{M}{\Delta}}{g-\alpha}} \approx \sqrt{I_{sat}}\sqrt{1-\frac{\alpha}{g}}\left(1+\frac{f(x)M}{2\Delta(g-\alpha)}\right)$$

The small change in the amplitude is therefore

$$\Delta A = \frac{M}{2\Delta(g-\alpha)}\sqrt{I_{sat}}\sqrt{1-\frac{\alpha}{g}}f(x)$$

In our system, for the range of parameters that we study, we find $f(x) \sim \frac{2MG}{\Delta}\cos^2(x)$ because $\frac{2GM}{D} = 1.347 \cdot 10^9 \left[\frac{rad}{sec}\right] \gg \Delta$, so that

$$\Delta A = \frac{G}{(g-\alpha)}\left(\frac{M}{\Delta}\right)^2\sqrt{I_{sat}}\sqrt{1-\frac{\alpha}{g}}\cos^2(x) \ .$$

First, we clearly see the doubled periodicity that comes from the square of the trigonometrical function, which is what we also observe in simulations. Moreover, the derivative of the simulated amplitude is proportional to the small signal

$$\frac{A'}{A} \sim \frac{G}{(g-\alpha)}\left(\frac{M}{\Delta}\right)^2 \sim 10^{-5}$$

, which means that our assumption of a small intensity deviation was reasonable.

5. Coherence of the final states

To retrieve the spectral phase and its coherence, we use SWIFTS measurement, a methods that enables phase detection with a fast photodetector [2]. [3] shows and explains the optical set-up and data acquisition scheme for the SWIFTS measurement. The main part of the setup is an interferometer designed in a folded Mach-Zehnder configuration whose purpose is to minimize back-reflections into the laser. The collimated laser beam of the investigated semiconductor laser is directed at the input port of the interferometer. At the interferometer's output, a fast Quantum Well Infrared Photodetector (QWIP) is used, from which both the DC and RF traces can be read simultaneously. A reference laser is used to acquire the optical path delay from the moving mirror. The spectrally resolved RF signal of the fast detector is down-mixed to ≈ 20 MHz to fall in the bandwidth of the Lock-In amplifier from which are obtained the Q and I quadrature components, required to retrieve the phase at each frequency. The quadrature components are the results of 10 coherent averages. The device was operated at a temperature of -20°C and a current ≈ $2I_{th}$. For the presented case, the injected RF power was +35 dBm.

We obtain the spectral phase from the averaged quadrature components, and show the spectrum and spectrally resolved phase profile in Fig. S2. The phase profile of the off-resonant case shows the feature of an FM comb behavior with phase jumps of ≈ π/2. SWIFTS technique also allows the measurement of the coherence between two adjacent modes and the results obtained are shown in Fig. S2d. The values ~1 indicate of high degree of coherence between all the modes. Since the studied device has a low optical output power, a small number of modes suffered a poor signal-to-noise ratio thus resulting in significant uncertainty in the coherence measurement. The shaded area surrounding each curve indicates the standard deviation obtained after averaging 10 spectra.

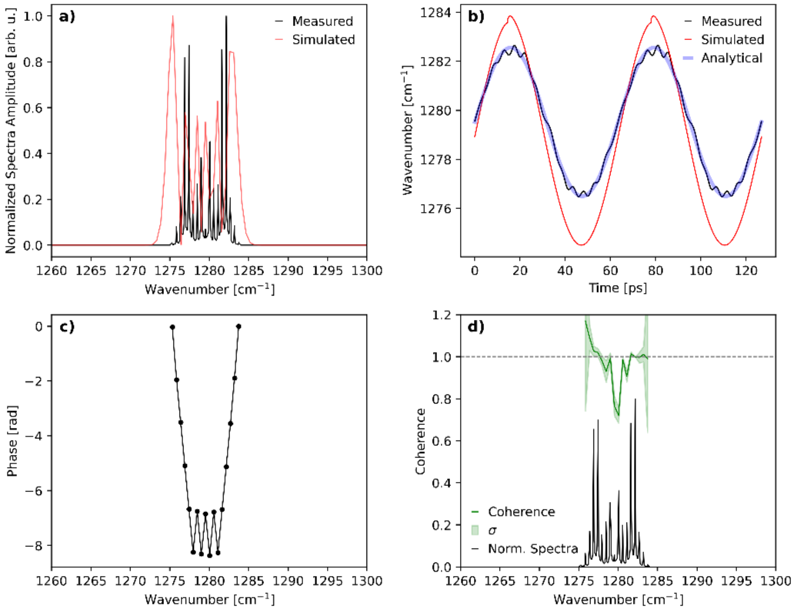

Figure S 2. Coherence of the measured state. (a) Measured averaged spectrum for a stable system for $\Delta f = 15.762GHz$ and its reconstruction for general parameters. (b) Retrieved instantaneous frequency $f_{inst} = \dot{\phi}$ with an analytical estimation and a simulation calculation. (a) and (b) indicate that the spectrum follows a Bessel function. (c) Measured spectral phase. (d) Spectrally resolved coherence plots with their standard deviation computed with 10 data sets.

6. Experimental setup

In this work, we specifically refer to the time resolved measurement in the falling edge of our switch that appears in Fig. 4 in the main text. We measured a 137ns fall time using a Mini-Circuits power detector ZX47-50-S+ with inverted voltage. This detector has a typical rise time of around 10ns whereas the typical fall time is very long, around 400ns. This indicates that the long fall time of the switching measurement is mainly caused by the power detector and not only by the switch, with the actual switching time being much shorter. To verify that the actual switching time doesn't have substantial influence on the fast quenching and the final state, we performed simulation of the EM equation in the laser (Fig. S3, left). The simulation shows a good agreement with the results presented in Fig. 4 of the main text, implying that the non-vanishing switching off time doesn't play a substantial role in the coherent decay to $\delta(\mathrm{m})$. To compare this result to linear or slow gain systems, we perform a simulation with the same conditions but after quenching at t_0 we turn off the fast-gain, see Fig. S3, right. Without coupling or interaction, the dynamics are governed by dissipative mechanisms only.

All details related to the processing, mounting and measurement of our semiconductor laser device are given in the supplementary information of ref [3].

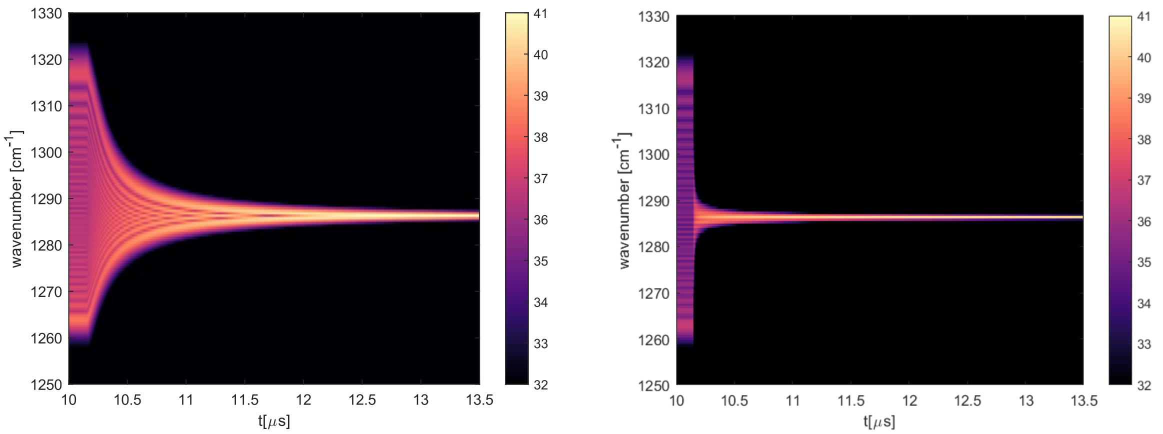

Figure S 3. Simulation of quenching a coupled system to a trivial lattice (uncoupled). At time $t_0 = 10.15\mu s$ we instantly switch off the modulation. This left plot reproduces the measurement captured in Fig. 4a of the main text. The good agreement implies that the switching off time measured by the slow power detector of 137ns doesn't play a substantial role in the coherent decay to $\psi(m, t \to \infty) = \delta(m)$. For comparison, we performed the same quench, but after t_0 we removed the fast-gain mechanism, leaving the system to evolve with a linear non-Hermitian propagator. It is clear that without interaction nor coupling, the governing mechanism is the gain curvature, which prevent complicated coherent dynamics of the wavefunction.

7. Interplay between complex dispersion, artificial electric field and fast gain

Our system is described well in synthetic space, and there are various elements that contribute to the dynamics. First, our system is RF modulated with detuning from resonance, therefore, we expect to have a static electric field in the synthetic space Δ, that eventually leads to oscillations. We indeed observe oscillation, as our model predicts (Figs. 2C,3A,S4). Second, dispersion in the time domain translates into potential energy or a non-uniform constant field. This leads to the presence of bound modes in the system (Fig. 1D, red), which are both standing waves in the synthetic and its reciprocal space k_m (which is actually the intracavity space z). Due to the limitation of the maximum kinetic energy $E_k = 4C$ (a direct result of the coupling between discrete states), the system also has modes that are bound to the onsite potential energy, $E \sim Dm^2$, with an extent proportional to E_k. These modes appear extended in the intracavity space with characteristic degenerate couples that are able to produce propagation in both directions (Fig. 1C in the main text, light and dark blue). These are the modes that we study here. We use $C = 1.23 \cdot 10^8 \frac{rad}{sec}$ and $D_{exp} = 1.75 \cdot 10^5 \frac{rad}{sec}$, with $G = 9.5462 \cdot 10^5 \frac{rad}{sec}$.

The third element is the gain curvature, which acts as a dissipative mechanism that extinguishes fluctuations at high frequencies. In the synthetic space analogy, each site would experience local loss, where the losses are spread quadratically along the synthetic lattice. In the case of the coupled system, the gain curvature sets different lifetimes for the supermodes, and consequentially the final mode at stabilization.

The fourth is the fast gain, dictating that approximately, at all times, the signal in time should maintain a quasi-constant intensity. This mechanism is local in real space but induces infinitely long interactions in synthetic space. This is the most important and unique feature of this work, and it contributes a novel type of interaction that repel high occupation in the k_m space, forcing the state to occupy equally the full reciprocal space, or in a translationally invariant system, the Brillouin zone.

We simulate Eq. 1 of the main text with all elements mentioned above. We reproduce the measurement results in Fig. 3 with their detuning values with excellent agreement and thereby acquire the parameters of the system (Fig. S4A). We also calculate the decay rate of the oscillations for different values of detuning and dispersion, and find that these time are between $0.2 - 1\mu s$ (Fig. S4B).

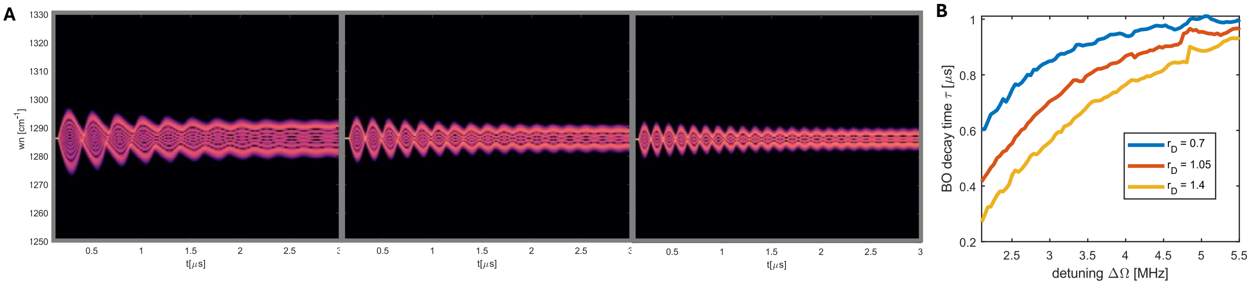

Figure S 4. Bloch oscillations and decay in simulations. (A) Simulation that reproduces the oscillations and decay that were experimentally measured and presented in Fig. 3 of the main text for values $\Delta f = 4.12MHz, 6.12MHz, 8.12MHz$, respectively.(B) Calculated decay rate of the oscillations for different values of detuning and dispersion.

Next, we present simulation results in Figs. S5 and S6 and compare the impact of the contribution of different emulation elements separately. First, we show that in the case of slow gain, dispersion is causing decoherence and therefore the Bloch oscillations are lost (Fig. S5B). Moreover, in case of fast gain and gain

curvature, the quadratic dispersion can be neglected at high detuning regimes (Fig. S5C). When dispersion is present, but gain curvature is 1000 times weaker than in the experiment, the spectrum still decays to a single Wannier stark dates in a coherent process (Fig. S5D). Finally, Fig. S6 shows that when there is no dispersion and gain curvature is very weak, the oscillation of the fast gain maintain coherence (Fig. S6A), while the oscillation in the slow gain regime substantially loose their shape through evolution (Fig. S6B), implying that slow gain lasers are not in a liquid phase. This phenomenon is even clearer through the observation of the intracavity intensity, which is analogous to band population. The fast gain is populating the full band throughout the emulation (Fig. S6C), while the slow gain is condensing to specific regions of the band (Fig. S6D).

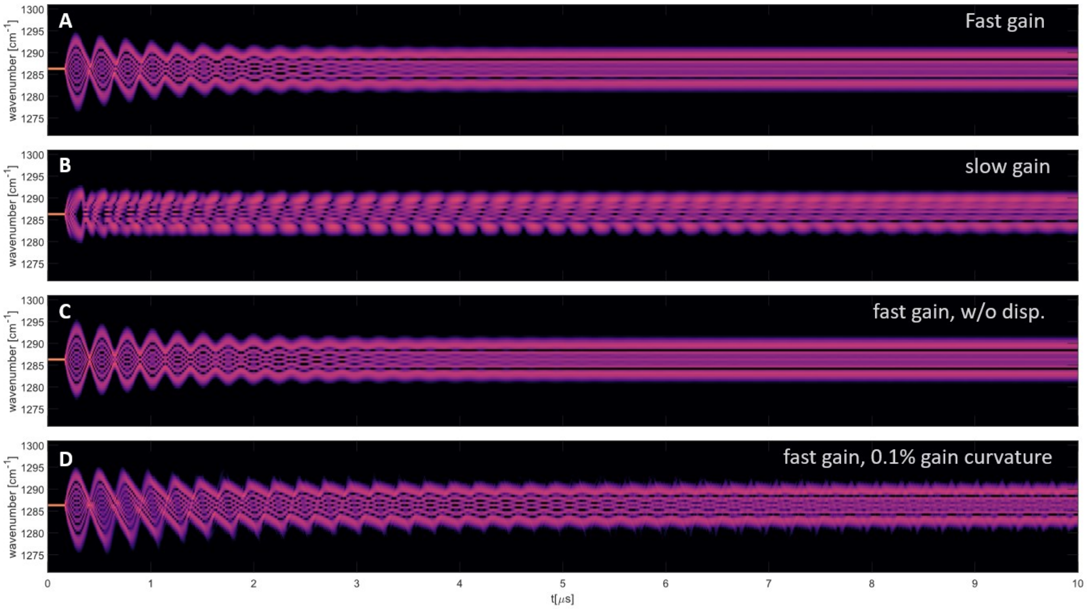

Figure S 5. Simulations of the dynamics under the influence of the different elements. (A) A simulation that reproduces a measurement in our system. (B) same as (A) but with slow gain. Due to dispersion we observe decoherence and therefore the Bloch oscillations are lost . (C) same as (A), but without quadratic dispersion. The simulation shows a results very similar to the measured data, implying that at this regime where $\Delta = 2\pi \cdot 4.12MHz > \Delta_c$, the quadratic dispersion is not playing a major role in the dynamics. (D) same as (A) but with gain curvature 1000 times smaller than in the experiment, showing that the decay to a Wannier Stark state is mainly induced by complex dispersion and the fast gain that strives for flat intensity, achieved through a single eigenmode with flat amplitude.

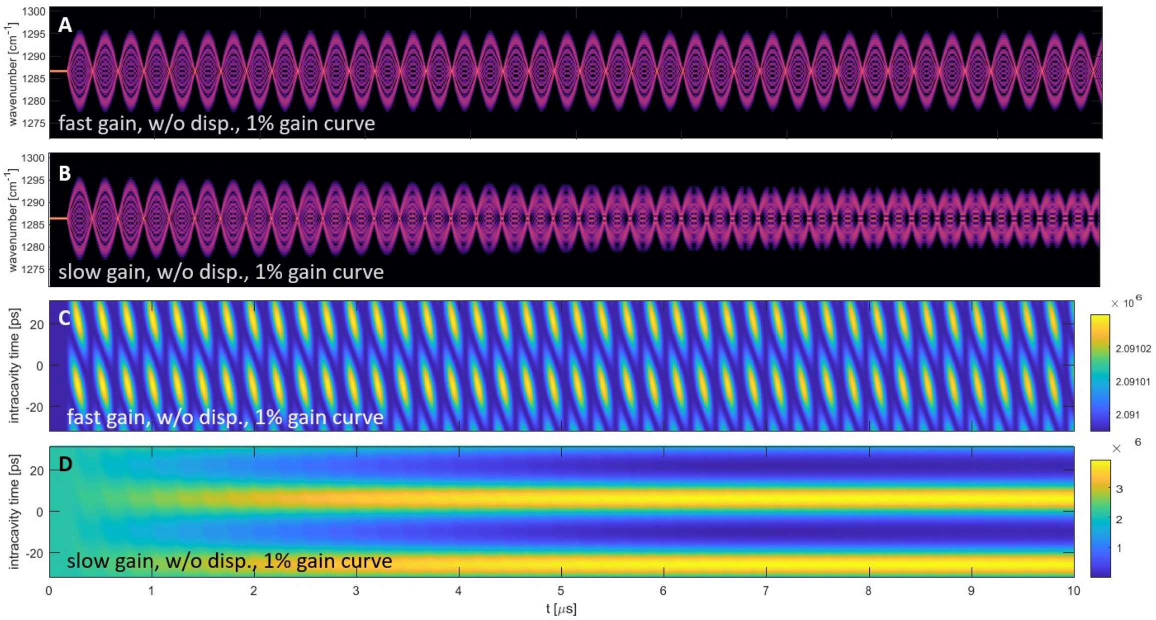

Figure S 6. Simulations of the dynamics when dispersion is absent. (A) same as Fig. S5A, but without any dispersion and gain curvature 100 time smaller than the experiment. The Bloch oscillation ideally persist infinitely, occupying equally the Brillouin zone through the time of the simulation. (B) same as (A) but with slow gain. The gain curvature is not protected by the fast gain, leading the higher modes to decay, and, in practice, ruining the oscillation. (C) the amplitude of the field in the cavity as a function of time of the parameters in (A). We observe very small fluctuations of the amplitude, smaller than 0.002%, which translates to constant full population of the Brillouin zone. (D) the amplitude of the field for slow gain, showing condensation in the effective Brillouin zone.

8. Perturbation analysis

To study the nonlinearity of the fast gain, we study perturbatively the following equation of the EM field in the cavity:

$$\dot{E} = \frac{1}{2}\left[g_0\left(1 - \frac{I}{I_s}\right) - \alpha_w\right]\left(\frac{c}{n}\right)E + i\frac{1}{2}\beta\nabla^2 E + \frac{1}{2}g_c\nabla^2 E - iMcos(Kz - \Delta t)E$$

We denote the steady state as E_0 and the perturbation as F. The gain saturation term become

$$\left[g_0\left(1 - \frac{|E_0|^2 + E_0F^* + E_0^*F + |F|^2}{I_s}\right) - \alpha_w\right](E_0 + F)$$
$$\approx \left[g_0\left(1 - \frac{|E_0|^2}{I_s}\right) - \alpha_w\right]E_0 - g_0\frac{E_0^2F^*}{I_s} + \left[g_0\left(1 - \frac{2|E_0|^2}{I_s}\right) - \alpha_w\right]F$$

The equation for the perturbation turns to

$$\dot{F} \sim -\frac{1}{2}g_0\left(\frac{c}{n}\right)\frac{E_0^2}{I_s}F^* + \frac{1}{2}\left[g_0\left(1 - \frac{2|E_0|^2}{I_s}\right) - \alpha_w\right]\left(\frac{c}{n}\right)F + i\frac{1}{2}\beta\nabla^2 F + \frac{1}{2}g_c\nabla^2 F - iMcos(x)F$$

, and for fully clamped gain ($g_0\left(1 - \frac{|E_0|^2}{I_s}\right) - \alpha_w \approx 0$), we get

$$\dot{F} \sim -\frac{1}{2}g_0\left(\frac{c}{n}\right)\frac{E_0^2}{I_s}F^* - \frac{1}{2}g_0\left(\frac{c}{n}\right)\frac{|E_0|^2}{I_s}F + i\frac{1}{2}\beta\nabla^2 F + \frac{1}{2}g_c\nabla^2 F - iMcos(x)F$$

The first term is inducing losses with coupling to the conjugate wave $\propto -E_0^2F^*$, and the second term is onsite loss. The rest describes a wavefunction trapped in a cosine potential with gain curvature. We assume the intensity of the steady state solution is I_0

$$\dot{F} \sim -\frac{1}{2}g_0\left(\frac{c}{n}\right)\frac{I_0}{I_s}\left(F + F^*e^{i2\phi(Kz-\Delta t)}\right) + i\frac{1}{2}\beta\nabla^2 F + \frac{1}{2}g_c\nabla^2 F - iMcos(Kz - \Delta\Omega t)F$$

, which is a general result in our system. Now, we can substitute E_0 with the analytical solution we found, and get to the following equation

$$\dot{F} \sim -\frac{1}{2}g_0\left(\frac{c}{n}\right)\frac{I_0}{I_s}\left(F + F^*e^{i2\frac{M}{\Delta\Omega}sin(Kz-\Delta t)}\right) + i\frac{1}{2}\beta\nabla^2 F + \frac{1}{2}g_c\nabla^2 F - iMcos(Kz - \Delta\Omega t)F$$

,with estimated parameters from experiments, $g_0 = 8.55[cm^{-1}], n = 3.3, I_0 = 4.4 \cdot 10^{12}\left[\frac{V}{m^2}\right], I_s = 8.2 \cdot 10^{12}\left[\frac{V}{m^2}\right]$, we find that the decay time is

$$\tau_F = \frac{nI_s}{g_0cI_0} = 24ps < T_{cycle} = 63ps \ll \frac{1}{C} = 8ns$$

Which is shorter than the roundtrip time, and much shorter than the tunneling time between modes, Indicating that this is the fastest process in our system. Additionally, the gain curvature is adding mode number dependent decay to the system (with $K = 1.09 \cdot 10^3$):

$$\tau_{gc,m} = (g_c m^2 \mathrm{K}^2)^{-1} = 0.52\mu s/m^2$$

For example, only from the 91^h mode the decay time will be faster than the cycle time, i.e. $\tau_{gc,91} = 62.8ps < T_{cycle} = 63ps$.

To study the impact of the fast decay of intensity fluctuations on the stability of the system, we performed simulations where we inject white noise to the lasers and measure its variation around the solution in the absence of noise [4]. In Fig. S7, we present the calculated the intensity variance $Var(t,N) = \left\langle \left(I_N(z,t) - I_c(z,t)\right)^2 \right\rangle_z$, where N is the noise level, z is the coordinate in the corotating frame, $I_c(z,t)$ and $I_N(z,t)$ are the time evolution of the intensity in the absence and presence of Noise with level N, respectively. The amount of noise intensity added in a single cycle is $N \cdot I_0$. In the two different cases of fast and slow gain, we observe a very different impact. The phase of the light when gain is slow does not support sufficient suppression of fluctuations, where noise destabilizes the system, generating large variations – which is especially seen when oscillations are present. With slow gain and low noise levels (0.01%), the largest variation occurs right after the moment of the quench ~160ns-500ns, where the state becomes more stable as the oscillations disappear. On the contrary, the fast gain doesn't experience any significant variation arising from the dynamics state, with noise levels that follow only the injected noise.

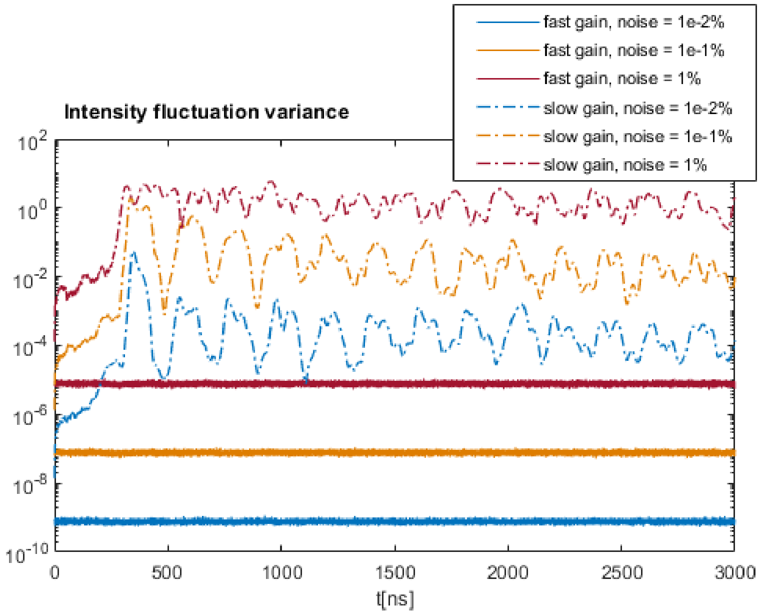

Figure S 7. Simulation of the fluctuations during a quench in the system. Calculated fluctuation variance presented as $Var(t,N) = \left\langle \left(I_N(z,t) - I_c(z,t)\right)^2 \right\rangle_z$ for fast (top) and slow (bottom) gain, shown for different values of noise in percentage from the average intensity. It is evident that the fast gain is superior in stability, being orders of magnitude more stable.

9. Dispersion of perturbations

To study the properties of the liquid of light in our system, we investigate the dispersion of the fluctuations. We use the following mode expansion $F(Kz - \Delta\Omega t) = \sum f_n e^{-in(Kz-\Delta\Omega t)}$ and write the equation of the perturbation derived in the previous section as

$$\begin{aligned}\sum \dot{f_n} e^{-in(Kz-\Delta\Omega t)} + \sum f_n e^{-in(Kz-\Delta\Omega t)}(in\Delta\Omega) \\ = -\frac{1}{2} g_0 \left(\frac{c}{n}\right)\frac{I_0}{I_s}\left(\sum f_n e^{-in(Kz-\Delta\Omega t)} + \sum f_n^* e^{in(Kz-\Delta\Omega t)} e^{i2\frac{M}{\Delta\Omega} sin(Kz-\Delta\Omega t)}\right) \\ + i\frac{1}{2}\beta\nabla^2 \sum f_n e^{-in(Kz-\Delta\Omega t)} + \frac{1}{2} g_c \nabla^2 \sum f_n e^{-in(Kz-\Delta\Omega t)} \\ - i\frac{M}{2}\sum f_n\left(e^{-i(n+1)(Kz-\Delta\Omega t)} + e^{-i(n-1)(Kz-\Delta\Omega t)}\right)\end{aligned}$$

We multiply by $e^{im(Kz-\Delta\Omega t)}$ and integrate to get

$$\begin{aligned}\dot{f_m} + in\Delta\Omega f_m = -\frac{1}{2} g_0 \left(\frac{c}{n}\right)\frac{I_0}{I_s}\left(f_m + \sum f_n^* \int dx e^{i\left((n+m)x + \frac{2M}{\Delta\Omega} sin(x)\right)}\right) - i\frac{1}{2}\beta m^2 K^2 f_m - \frac{1}{2} g_c m^2 K^2 f_m \\ - i\frac{M}{2}(f_{m+1} + f_{m-1})\end{aligned}$$

The result of the last integral is simply a Bessel function, $\frac{1}{2\pi}\int dx e^{i(nx+\alpha sin(x))} = J_n(-a)$, so we get

$$\dot{f_m} = -\alpha\left(f_m + \sum f_l^* J_{(l+m)}\left(-\frac{2M}{\Delta\Omega}\right)\right) + (-iDm^2 - i\Delta\Omega m - G_c m^2) f_m - i\frac{M}{2}(f_{m+1} + f_{m-1})$$

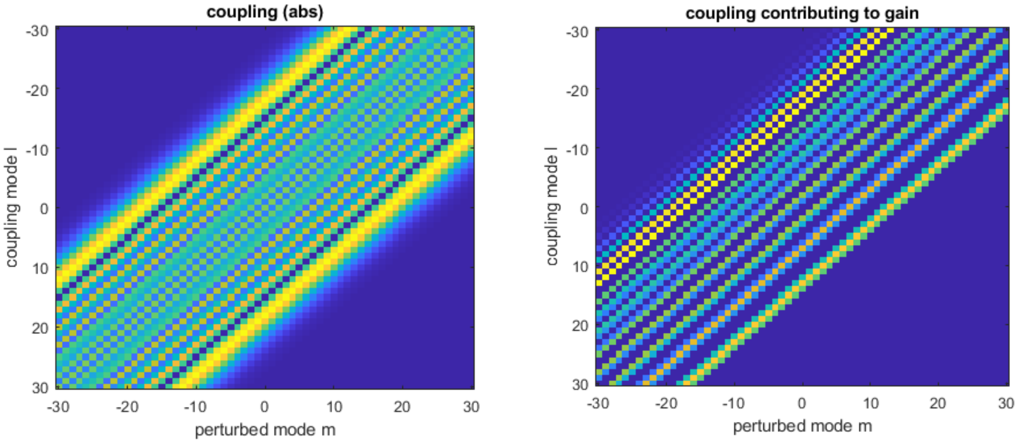

Figure S 8. The absolute value (left) and the positive part (right) of the dissipative coupling in the perturbation equation.

Due to the coupling to the conjugate terms, we divide the equation into real and imaginary parts.

We use the form $f_m = x_m + iy_m$, so that

$$\begin{aligned}\dot{x_m} + i\dot{y_m} = -\alpha\left(x_m + iy_m + \sum (x_l - iy_l) J_{(l+m)}\left(-\frac{2M}{\Delta\Omega}\right)\right) + (-iDm^2 - i\Delta\Omega m - G_c m^2)(x_m + iy_m) \\ - i\frac{M}{2}(x_{m+1} + iy_{m+1} + x_{m-1} + iy_{m-1})\end{aligned}$$

Next, we divide it to the real and imaginary parts (see Fig. S8), to find the equations of the dynamics

$$\dot{x_m} = -\alpha\left(x_m + \sum x_l J_{(l+m)}\left(-\frac{2M}{\Delta\Omega}\right)\right) - G_c m^2 x_m + (Dm^2 + \Delta\Omega m)y_m + \frac{M}{2}(y_{m+1} + y_{m-1})$$

$$\dot{y_m} = -\alpha\left(y_m - \sum y_l J_{(l+m)}\left(-\frac{2M}{\Delta\Omega}\right)\right) - G_c m^2 y_m - (Dm^2 + \Delta\Omega m)x_m - \frac{M}{2}(x_{m+1} + x_{m-1})$$

We calculate the dispersion and eigenmodes using the parameters of our system, and present the results in Fig. S9. The modes of the fluctuations around the central mode decay relatively slow, with decay rates around 22ns, which is around 3 times slower than the coupling. This is a typical behaviors of fluctuations in lasers that match the lasing regime.

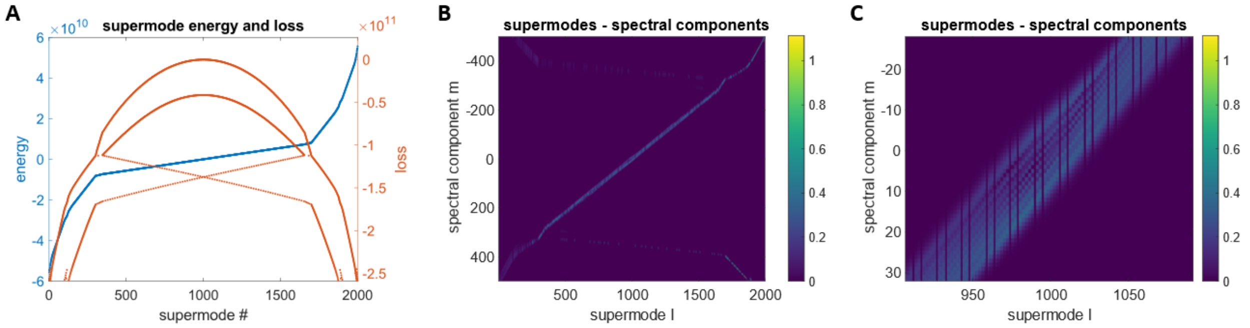

Figure S 9. Dispersion and supermodes of the fluctuations. Using the equation for the fluctuations, we calculate the supermodes supported in the system. (A) shows the dispersion and losses of these modes. Some modes have very little losses, with the longest decay time of ~22ns. (B) supermodes of the system form a ladder similar to Wannier stark, where (C) is zooming on the modes with the longest lifetime.

References

[1] L. Yuan and S. Fan, Bloch oscillation and unidirectional translation of frequency in a dynamically modulated ring resonator, Optica, Vol. 3, Issue 9, Pp. 1014-1018 **3**, 1014 (2016).

[2] D. Burghoff, Y. Yang, D. J. Hayton, J.-R. Gao, J. L. Reno, and Q. Hu, Evaluating the coherence and time-domain profile of quantum cascade laser frequency combs, Opt Express **23**, 1190 (2015).

[3] I. Heckelmann, M. Bertrand, A. Dikopoltsev, M. Beck, G. Scalari, and J. Faist, Quantum walk comb in a fast gain laser, Science (1979) **382**, 434 (2023).

[4] E. G. Turitsyna, S. V Smirnov, S. Sugavanam, N. Tarasov, X. Shu, S. A. Babin, E. V Podivilov, D. V Churkin, G. Falkovich, and S. K. Turitsyn, The laminar–turbulent transition in a fibre laser, Nat Photonics **7**, 783 (2013).
